# Understanding the roles and regulation patterns of circRNA on its host gene in tumorigenesis and tumor progression

**DOI:** 10.1186/s13046-023-02657-6

**Published:** 2023-04-15

**Authors:** Jianxia Wei, Mengna Li, Changning Xue, Shipeng Chen, Lemei Zheng, Hongyu Deng, Faqing Tang, Guiyuan Li, Wei Xiong, Zhaoyang Zeng, Ming Zhou

**Affiliations:** 1 NHC Key Laboratory of Carcinogenesis, Hunan Key Laboratory of Oncotarget Gene, Hunan Cancer Hospital and the Affiliated Cancer Hospital of Xiangya School of Medicine, Central South University, Changsha, 410013 China; 2 Cancer Research Institute, Central South University, Changsha, 410078 China; 3 The Key Laboratory of Carcinogenesis and Cancer Invasion of the Chinese Ministry of Education, Central South University, Changsha, 410078 China

**Keywords:** CircRNA, Host gene, Regulation, Tumorigenesis, Tumor progression

## Abstract

Circular RNAs (circRNAs) are a novel type of endogenous non-coding RNAs, which are covalently closed loop structures formed by precursor mRNAs (pre-mRNAs) through back-splicing. CircRNAs are abnormally expressed in many tumors, and play critical roles in a variety of tumors as oncogenes or tumor suppressor genes by sponging miRNAs, regulating alternative splicing and transcription, cis-regulating host genes, interacting with RNA binding proteins (RBPs) or encoding polypeptides. Among them, the regulation of circRNAs on their corresponding host genes is a critical way for circRNAs to exit their functions. Accumulating evidence suggests that circRNAs are able to regulate the expression of host genes at the transcriptional level, post-transcriptional level, translational level, post-translational level, or by encoding polypeptides. Therefore, this paper mainly summarized the roles and association of circRNAs and their corresponding host genes in tumorigenesis and tumor progression, generalized the circRNAs that function synergistically or antagonistically with their host genes, and elaborated the mechanisms of mutual regulation between circRNAs and their host genes. More importantly, this review provides specific references for revealing the potential application of circRNAs combined with their host genes in tumor diagnosis, treatment and prognosis.

## Background

Increasing evidence on non-coding RNAs (ncRNAs) has revealed their critical roles in tumorigenesis [1]. Circular RNAs are a novel type of non-coding RNAs, which are covalently closed loop structures formed by back-splicing through different mechanisms [2, 3]. Most human exonic circRNAs are less than 1500 nt in length, with a median length of around 500 nt [4]. In recent years, circular RNAs have become a new hotspot in the field of non-coding RNAs. With the development and improvement of deep sequencing and bioinformatics methods, the biogenesis and function of circRNAs have been widely studied. More importantly, clinical data showed that the expression of circRNAs was different in a variety of diseases, including tumors, suggesting that circRNAs have regulatory roles in carcinogenesis and tumor progression [5–11]. Additionally, the functions and mechanisms of circRNAs involved in different tumors may be rather diverse. Increasing evidence shows that circRNAs exert their oncogenic or tumor suppressor roles by acting as miRNA sponges, binding to RNA-binding proteins (RBPs), regulating alternative splicing or transcription, encoding peptides, regulating the expression of host genes, or acting as exosomal circRNAs [12, 13]. Among them, the regulation of circRNAs on their corresponding host genes is a critical mechanism for their function. Increasing studies have clarified that circRNAs can participate in tumor progression by positively or negatively regulating the expression and function of their host genes. Moreover, circRNAs are highly stable compared with linear RNAs, resistant to RNase R, and have tissue and cell specificity and high abundance, so circRNAs can be detected in human body fluids such as plasma and saliva [14, 15]. Therefore, targeting circRNAs and their host genes might be novel strategies for early diagnosis, effective treatment and prognostic evaluation of tumors [16–19].

In this review, we summarized the roles and association of circRNAs and their host genes in tumorigenesis and tumor progression, generalized the circRNAs that function synergistically or antagonistically with their host genes, and elaborated the mechanisms of mutual regulation between circRNAs and their host genes. We also discussed the clinical potential of circRNAs combined with their host genes in tumor diagnosis, treatment and prognosis as biomarkers and therapeutic targets.

## Functional relationship between circRNAs and their host genes

### CircRNAs that are functionally consistent with their host genes

Circular RNAs, which are derived from their corresponding host genes [16–20], have been proved to be important regulators of human tumors. In addition, according to previous studies, most circRNAs have been found to have the same functions as their host genes and play synergistic roles in tumors, of which some of them are highly expressed in tumor tissues and can promote the expression of the host genes through a variety of mechanisms, thus to promote tumor proliferation, migration, invasion, stemness, drug resistance and radiation resistance of tumor cells as oncogenes, such as circ-EGFR [21], circ-ENO1 [22] and circ-Amotl1 [23, 24]. While other of them are lowly expressed in tumor tissues, which can continuously activate their host genes and inhibit the malignant phenotype of tumors as tumor suppressors, such as circ-Foxo3 [16, 25], circ-ITCH [26–28] and circ-FBXW7 [29, 30]. All of the circRNAs that function synergistically with their host genes in tumor tissues or cells were generalized based on their expression patterns and functions, as shown in Table 1 [16–19, 21–79]. As circRNA is derived from its host gene, the host gene always promotes the formation and expression of its circRNA, therefore, the circRNA/host gene regulation axis could form a positive feedback loop to synergistically play a critical role in tumorigenesis and tumor progression.

**Table 1:** CircRNAs that are functionally consistent with their host genes

Circular RNA	Host gene	Expression	Cancer type	Potential function	Mechanism	Reference
circ-EGFR	EGFR	Up	Glioblastoma	Promote cell proliferation and tumor growth	Formed a protein-complex, termed rtEGFR, which interacted with EGFR, maintained EGFR membrane localization and attenuated EGFR endocytosis and degradation	[21]
circ-ENO1	ENO1	Up	Lung adenocarcinoma	Promote glycolysis, proliferation, migration and EMT; inhibit apoptosis	circ-ENO1/miR-22-3p/ENO1	[22]
circ-Amotl1	Amotl1	Up	Breast cancer; cervical cancer	Promote proliferation, migration, epithelial-mesenchymal transition (EMT) and tumor growth in vivo; decrease apoptosis	circ-Amotl1/c-myc (nuclear translocation and stability); Circ-Amotl1/miR-485-5P/Amotl1	[23, 24]
circCOL6A3	COL6A3	Up	Gastric cancer	Promote cell proliferation and migration; inhibit apoptosis	circCOL6A3/miR-3064-5p/COL6A3	[31]
circ_MMP2	MMP2	Up	Hepatocellular carcinoma	Promote metastasis of hepatocellular carcinoma in vitro and in vivo	circ_MMP2/miR-136-5p/MMP2	[32]
circDnmt1	Dnmt1	Up	Breast cancer	Promote proliferation and tumor xenograft growth; stimulate cellular autophagy; inhibit cellular senescence	circDnmt1/p53 and AUF1 (nuclear translocation and transcription)/Dnmt1	[33]
circXIAP (circ0005276)	XIAP	Up	Prostate cancer	Promote proliferation, migration and EMT	circXIAP/FUS/XIAP	[34]
FECR1	FLI1	Up	Breast cancer	Promote tumor metastasis	FECR1/recruit TET1and DNMT1/FLI1	[35]
circ-CUX1	CUX1	Up	Neuroblastoma (NB)	Promote aerobic glycolysis, tumorigenesis, and aggressiveness of NB cells	circ-CUX1/EWSR1/MAZ/CUX1	[36]
circβ-catenin	β-catenin	Up	Liver cancer	Promote cell growth and migration and tumorigenesis and metastasis in vivo	β-catenin-370aa/GSK3β/β-catenin/Wnt/β-catenin pathway	[37]
circFNTA	FNTA	Up	Bladder cancer	Promote cell invasion and cisplatin chemo-resistance; promote tumor growth and metastasis in vivo	AR/ADAR2/circFNTA/miR-370-3p/FNTA/KRAS	[38]
circGFRA1	GFRA1	Up	Triple negative breast cancer	Promote cell proliferation and tumor growth in vivo; inhibit apoptosis	circGFRA1/miR-34a/GFRA1	[39]
circ-0075804	E2F3	Up	Retinoblastoma	Promote cell proliferation and suppress cell apoptosis	circ-0075804/HNPNPK/E2F3 (mRNA stability)	[40]
circ-CCND1 (hsa_circ_0023303)	CCND1	Up	Laryngeal squamous cell carcinoma	Promote cell proliferation in vitro and tumor growth in vivo	circ-CCND1/HuR and miR-646/CCND1	[41]
circ-MMP9 (hsa_circ_ 0001162)	MMP9	Up	Oral squamous cell carcinoma	Promote migration and invasion and lung metastasis in vivo	circ-MMP9/AUF1 and miR-149/MMP9	[42]
hsa_circ_0047905	SERPINB5	Up	Gastric cancer	Promote cell proliferation and invasion	Not mentioned	[43]
hsa_circ_0138960	GDA	Up	Gastric cancer	Promote cell proliferation and invasion	Not mentioned	[43]
hsa_circRNA7690-15	GDA	Up	Gastric cancer	Promote cell proliferation and invasion	Not mentioned	[43]
circ_0069765	KIT	Up	Gastrointestinal stromal tumors	Promote tumor oncogenesis and progression	circ_0069765/miR-142-5p, miR-144-3p and miR-485-3p/KIT (regulatory network)	[44]
circ_0084097	PLAT	Up	Gastrointestinal stromal tumors	Promote tumor oncogenesis and progression	circ_0084097/miR-142-5p, miR-144-3p and miR-485-3p/PLAT (regulatory network)	[44]
circ_0079471	ETV1	Up	Gastrointestinal stromal tumors	Promote tumor oncogenesis and progression	circ_0079471/miR-142-5p, miR-144-3p and miR-485-3p (regulatory network)	[44]
circ_0001730	EPHB4	Up	Glioblastoma	Promote cell growth, invasion and tumor growth in vivo	SP1/EPHB4/circ_0001730/miR-326/Wnt7B/β-catenin pathway	[45]
circCRIM1	CRIM1	Up	Ovarian cancer	Promote cell viability, migration, invasion and tumor growth in vivo; inhibit apoptosis	circCRIM1/miR-145-5p/CRIM1; encode 188aa protein; circCRIM1/miR-383-5p/ZEB2	[46]
circMETTL3	METTL3	Up	Breast cancer	Facilitate cell proliferation, migration, invasion and tumor growth in vivo	METTL3/circMETTL3/miR-31-5p/CDK1	[47]
circ-SIRT1	SIRT1	Up	Colorectal Cancer	Promote cell proliferation, invasion, and EMT	circ-SIRT1/EIF4A3/N-cadherin/vimentin	[48]
hsa_circ_0003141	UBAP2	Up	Hepatocellular carcinoma	Promote cell proliferation, invasion and tumor growth in vivo; inhibit apoptosis	hsa_circ_0003141/miR-1827/UBAP2	[49]
hsa_circ_0006401	col6a3	Up	Colorectal cancer	Promote cell proliferation, migration, invasion, tumor growth and metastasis in vivo; inhibit apoptosis	Encode a novel 198-aa functional peptide to promote stability of the host gene col6a3 mRNA	[50]
circ-SKA3	SKA3	Up	Medulloblastoma	Promote cell proliferation, migration and invasion	Not mentioned	[51]
circ-DTL	DTL	Up	Medulloblastoma	Promote cell proliferation, migration and invasion	Not mentioned	[51]
F-circEA1	EML4-ALK1	Up	Non-small cell lung cancer	Promote cell proliferation, migration, invasion and tumor growth in vivo; inhibit apoptosis; promote drug resistance to crizotinib	F-circEA1/EML4-ALK1/ALK downstream signaling pathway	[52]
circMET	MET	Up	Colorectal cancer	Promote cell proliferation and growth	circMET/miR-410-3p/MET	[53]
hsa_circ_0000069	STIL	Up	Pancreatic cancer	Promote cell proliferation, migration, invasion and growth of xenograft pancreatic cancer tumors in vivo; inhibit apoptosis	hsa_circ_0000069/miR-144/STIL/ Gli1	[54]
hsa_circ_0062270	CDC45	Up	Melanoma	Promote the viability, proliferation, invasion and tumor growth in vivo; inhibit apoptosis	hsa_circ_0062270/EIF4A3/CDC45	[55]
circ-NOTCH1	NOTCH1	Up	Gastric cancer	Promote cell migration, invasion, stemness, tumor growth and metastasis in vivo	circ-NOTCH1/miR-449c-5p/MYC/NOTCH1	[56]
circZEB1	ZEB1	Up	Prostate cancer	Decrease radiosensitivity	TR4-mediated QKI/circZEB1/miR-141-3p/ZEB1	[57]
circ3323	APP	Up	Bladder cancer	Promote cell invasion and migration	circ3323/miR-186-5p/APP	[58]
F-circAE2	AML1-ETO	Up	Leukemia cell	Promote cell proliferation in vitro and in vivo	F-circAE2/ENO-1	[59]
circBA9.3	BCR-ABL1	Up	Leukemia cell	Promote proliferation and inhibit apoptosis	Increase the protein expression of its host gene BCR-ABL1	[60]
F-circSRs (F-circSR1, F-circSR2)	SLC34A2-ROS1	Up	Non-small cell lung cancer	Promote cell migration	F-circSRs/miR-150-5p, miR-194-3p and miR-515-5p	[61]
F-circM9	MLL-AF9	Up	Leukemia cell	Promote cell proliferation and clonogenicity	Not mentioned	[62, 63]
circ-Foxo3	Foxo3	Down	Non-small cell lung cancer (NSCLC); Breast cancer	Inhibit cell proliferation, migration and invasion of NSCLC cells; Inhibit proliferation and promote apoptosis of breast cancer cells	circ-Foxo3/miR-155/Foxo3; Foxo3P and circ-Foxo3/miR-22, miR-136*, miR-138, miR-149*, miR-433, miR-762, miR-3614–5p and miR-3622b–5p/Foxo3	[16, 25]
circ-ITCH	ITCH	Down	Lung cancer; esophageal squamous cell carcinoma; colorectal cancer; bladder cancer	Inhibit cell proliferation of lung cancer; Inhibit cell proliferation and tumor growth in vivo; Inhibit cell proliferation of colorectal cancer; Inhibit proliferation, migration, invasion and metastasis of bladder cancer both in vitro and in vivo	Cir-ITCH/miR-7 and miR-214/ITCH/Wnt/β-catenin pathway; cir-ITCH/miR-7, miR-17 and miR-214/ITCH/Dvl2/Wnt/β-catenin pathway; Cir-ITCH/miR-7 and miR-20a/ITCH/Wnt/β-catenin pathway/c-myc and cyclinD1; circ-ITCH/miR-17, miR-224/p21, PTEN	[17, 26–28]
circ-FBXW7	FBXW7	Down	Glioblastoma; triple-negative breast cancer (TNBC)	Inhibit cell proliferation and the cell cycle of glioblastoma in vitro and in vivo; Inhibit cell proliferation and migration of TNBC, and tumor growth in vivo	FBXW7-185aa/USP28/c-Myc; circFBXW7/miR-197-3p/FBXW7 and FBXW7-185aa/USP28/FBXW7/c-Myc	[29, 30]
circITGA7	ITGA7	Down	Colorectal cancer	Inhibit cell proliferation and migration in vitro and tumor growth in vivo	circITGA7/miR-370-3p/NF1/Ras pathway/RREB1/ITGA7	[18]
circZKSCAN1	ZKSCAN1	Down	Hepatocellular carcinoma	Inhibit cell proliferation, migration, invasion and tumor growth in vivo	Not mentioned	[19]
ciRS-7/CDR1-AS	CDR1	Down	Glioblastoma multiforme	Inhibit cell proliferation	MiR-671-5p/CDR1-AS/CDR1/VSNL1	[65]
circ0006916	HOMER1	Down	Lung cancer	Inhibit cell proliferation	TNRC6A/circ0006916/miR-522-3p/PH domain and PHLPP1	[66]
circHIPK3	HIPK3	Down	Bladder cancer	Inhibit migration, invasion, and angiogenesis of bladder cancer cells in vitro and suppress bladder cancer growth and metastasis in vivo	circHIPK3/miR-558/heparinase (HPSE)	[67]
circ-SHPRH (hsa_circ_0001649)	SHPRH	Down	Glioblastoma; hepatocellular carcinoma (HCC)	Inhibit cell proliferation of glioblastoma and tumor growth in vivo; Inhibit proliferation and migration of HCC in vitro and in vivo	circ-SHPRH/SHPRH-146aa/SHPRH/PCNA; circ-0001649/miR-127-5p, miR-612 and miR-4688/SHPRH	[68, 69]
circ-AKT3	AKT3	Down	Glioblastoma	Inhibit cell proliferation, radiation resistance and in vivo tumorigenicity of glioblastoma cells	circ-AKT3/AKT3-174aa/AKT-thr308 phosphorylation/sequential activation by p-PDK1	[70]
circ-EPB41L5	EPB41L5	Down	Glioblastoma	Inhibit cell proliferation, migration, invasion and the growth of brain xenograft tumors of glioma cells	circ-EPB41L5/miR-19a/EPB41L5/ RhoC/p-AKT	[71]
hsa_circ_0099329	PPFIA2	Down	Glioblastoma	Not mentioned	Not Mentioned	[72]
circKCNN2	KCNN2	Down	Hepatocellular carcinoma	Inhibit HCC cell proliferation, colony formation, migration, and tumor formation in a mouse model	circKCNN2/miR-520c-3p/ methyl-DNA-binding domain protein 2 (MBD2)/FGFR4	[73]
circKEAP1	KEAP1	Down	Lung adenocarcinoma	Inhibit cell proliferation, migration, invasion and tumor growth in vivo	circKEAP1/miR-141-3p/ KEAP1/NRF2	[74]
circ_0018414	DKK1	Down	Lung adenocarcinoma	Inhibit cell proliferation, stemness and tumor growth in vivo; promote cell apoptosis	circ_0018414/ miR-6807-3p/DKK1/ Wnt/β-catenin	[75]
circASS1	ASS1	Down	Breast cancer	Inhibit invasion and migration	circASS1/ miR-4443/ASS1	[76]
circ-PTEN (hsa_circ_0094342)	PTEN	Down	Non-small cell lung cancer	Inhibit cell proliferation and tumor growth in vivo	DHX9/circ-PTEN/miR-155 and miR-330-3p/PTEN/PI3K/AKT pathway	[77]
hsa_circ_0036722	RHGG	Down	Laryngeal squamous cell carcinoma	Inhibit cell proliferation in vitro	hsa_circ_0036722/miR-1248/ RHCG	[78]
circ_LARP4	LARP4	Down	Ovarian cancer	Inhibit cell proliferation, invasion and migration	circ_LARP4/miR-513b-5p/LARP4	[79]

### CircRNAs with opposite functions to their host genes

As indicated in the above that most circRNAs have the same expression patterns as their host genes, however, some studies have found that the expression patterns of a few of circRNAs are opposite to that of their host genes, proving that some circRNAs have different functions from the linear products encoded by their host genes [6, 80–88]. For example, circGSK3β was upregulated in the tumor tissues compared to the normal tissues, which was confirmed to act as an oncogene to promote cell migration, invasion and EMT by inhibiting GSK3β/β-catenin signaling axis activity in esophageal squamous cell carcinoma (ESCC), while its host gene GSK3β presented low expression and tumor suppressor role in ESCC [80]. Meanwhile, Circ-Ccnb1 was found to bind to both Ccnb1 and Cdk1 proteins to dissociate the formation of the Ccnb1-Cdk1 complex and inhibit the tumor-promoting function of its host gene Ccnb1 by forming a large complex containing circ-Ccnb1, Ccnb1 and Cdk1, thereby inhibiting breast cancer cell proliferation, migration, invasion and tumor growth in vivo [83]. All of the circRNAs that function oppositely to their host genes in tumors were generalized based on their expression patterns and functions, as shown in Table 2. As the expression patterns of these circRNAs are different from that of their host genes, they antagonize the functions of their host genes and play negative feedback roles. Thus, circRNAs and their host genes constitute complex regulatory mechanisms and action networks of organisms [89].

**Table 2:** CircRNAs with opposite functions to their host genes

Circular RNA	Expression	Host gene	Expression	Cancer type	Potential function of circRNAs	Mechanism	Reference
circGSK3β	Up	GSK3β	Down	Esophageal squamous cell carcinoma (ESCC)	Promote ESCC cell migration, invasion and EMT	circGSK3β/GSK3β/β-catenin signaling	[80]
circPOK	Up	Zbtb7a	Down	Mesenchymal tumor	Regulate pro-proliferative and pro-angiogenic factors by co-activation of the ILF2/3 complex	circPOK/ILF2/3 complex/Il6 and Vegf	[6]
hsa_circ_0079993	Up	POLR2J4	Down	Colorectal cancer	Promote cell proliferation and tumor growth in vivo	circ_0079993/miR-203a-3p.1/CREB1	[81]
circ-HuR (hsa_circ_0049027)	Down	HuR	Up	Gastric cancer	Inhibit the growth, invasion, and metastasis of gastric cancer cells in vitro and in vivo	circ-HUR/CCHC-type zinc finger nucleic acid binding protein (CNBP)/HuR	[82]
circ-Ccnb1	Down	Ccnb1	Up	Breast cancer	Inhibit cell proliferation, migration, invasion and tumor growth in vivo	circ-Ccnb1 bind to both Ccnb1 and Cdk1 proteins to form a large complex to dissociate the formation of Ccnb1-Cdk1 complex and inhibit the tumor-promoting function of its host gene Ccnb1	[83]
circYap	Down	Yap	Up	Breast cancer	Inhibit cell proliferation, adhesion, migration and invasion	circYap/PABP and eIF4G/Yap	[84]
circPABPN1 (hsa_circ_0031288)	Down	PABPN1	Up	Cervical carcinoma	Inhibit cell proliferation	circPABPN1/HuR/PABPN1	[85]
circ-MYBL2	Down	MYBL2	Up	Multiple myeloma (MM)	Inhibit MM cell viability, DNA synthesis, cell cycle progression and tumor growth in vivo	circ-MYBL2/Cyclin F/MYBL2	[86]
circSMARCA5	Down	SMARCA5	Up	Breast cancer	Inhibit DNA damage repair function and enhance the cisplatin response in breast cancer in vitro and in vivo	circSMARCA5 formed R-loops with its host gene to terminate SMARCA5 transcription	[87]
circ_0004296	Down	ETS1	Up	Prostate cancer	Inhibit cell proliferation, migration, invasion, epithelial-mesenchymal transition, tumor growth and metastasis in vivo	circ_0004296/EIF4A3/ETS1	[88]

## Mechanisms of circRNAs regulating host genes

### Regulation at the transcriptional level

**Fig. 1:**
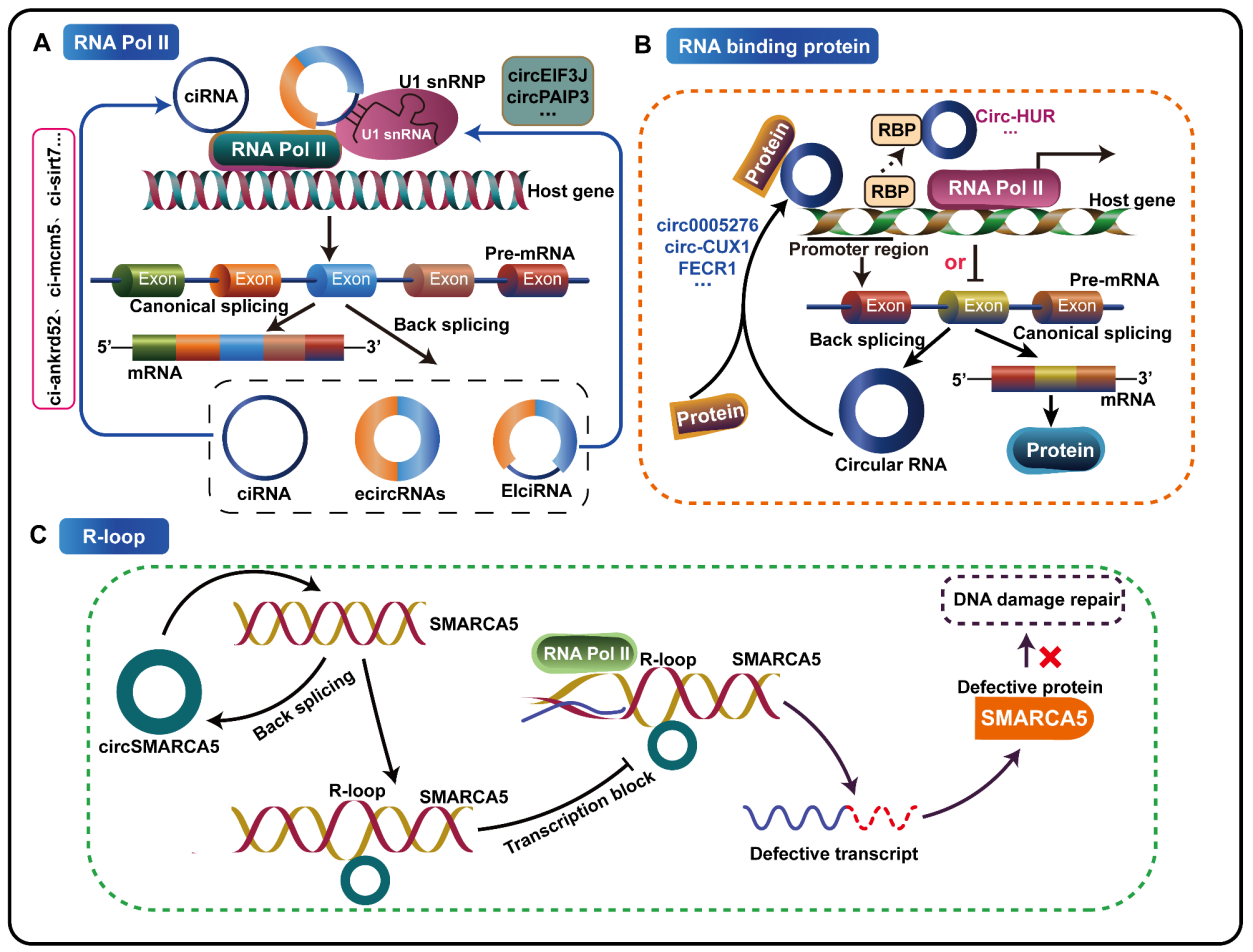
CircRNAs regulate their host genes expression at the transcriptional level. **A** CircRNAs can be classified into three subtypes: exonic circRNAs (ecircRNAs), intronic circRNAs (ciRNAs), and exon-intron circRNAs (EIciRNAs). Some ciRNAs and EIciRNAs can promote the transcriptional activity of their host genes by binding to RNA polymerase II (Pol II). **B** CircRNAs can function as protein decoys, scaffolds or recruiters to promote or inhibit their host genes expression. **C** Some circRNAs, such as circSMARCA5, can increase the cleavage efficiency of homologous exon-defective mRNA by forming R-loops, which in turn terminated transcription, and affect their host genes expression

Promoter regions are the most widely studied specific regions in transcriptional regulation [90, 91]. CircRNAs have been reported to positively or negatively regulate the transcription of their host genes by binding to RNA polymerase II (Pol II) [92, 93], recruiting proteins [34–36, 82], or by forming an R-loop [87, 94, 95] to target the transcriptional regulatory regions of their host genes (Fig. 1).

#### Promoting the transcriptional activity of their host genes by binding to poly II

According to the source and location of the circRNAs sequences, the currently discovered circRNAs can be classified into three subtypes, exonic circRNAs (ecircRNAs), intronic circRNAs (ciRNAs) and exon-intron circRNAs (EIciRNAs) [1, 96]. Detailed studies have clarified that some ciRNAs are mainly distributed in the nucleus and interact with RNA Pol II to regulate the transcriptional activity of host genes in a cis-acting manner. Ci-ankrd52, ci-mcm5 and ci-sirt7 have been reported to be mainly enriched in the transcription sites of their host genes, which are related to the transcription extension mediated by RNA Pol II and act as positive transcription regulators of host genes to enhance the expression of host genes [92]. Exon-intron circRNAs, which are formed by cyclization of RNAs with intron retention, are also enriched in the nucleus and associated with Pol II in human cells [93, 97, 98]. It was found that circEIF3J and circPAIP2, two exon-intron circRNAs, were able to interact with RNA polymerase II, U1 snRNP and host gene promoters to enhance the transcription of their host genes in a cis-acting manner by forming a positive feedback loop, and the deletion of these circRNAs reduced the transcription level of the corresponding EIF3J or PAIP2 host genes [93] (Fig. 1A).

#### Regulating the expression of their host genes by recruiting proteins

Many studies have reported that there are highly specific RNA-binding protein binding sites on circRNAs, therefore circRNAs can function as protein decoys, scaffolds and recruiters to recruit single or multiple proteins to the specific regions of the target promoter, thereby regulating transcription activation and expression of the host genes, which may also be an important mechanism for circRNAs participating in tumor progression [34–36, 82]. So far, these protein types have been found to include RNA-binding proteins (RBPs), DNA demethylase and DNA methyltransferase.

Some circRNAs were confirmed to transcriptionally activate the expression of their host genes and downstream target genes by recruiting proteins, thus promoting or inhibiting tumor progression (Fig. 1B). For example, Feng et al. determined that circ0005276 is a new circRNA formed by back-splicing of its host gene XIAP, which could recruit the RNA-binding protein FUS to the promoter region of the host gene XIAP and transcriptionally activate the expression of XIAP, thus promoting the occurrence and development of prostate cancer (PCa) [34]. Li et al. found that circ-CUX1, encoded by CUX1, is highly expressed in neuroblastoma and could bind to EWS RNA-binding protein 1 (EWSR1), thus promoting the interaction between EWSR1 and MYC-associated zinc finger protein (MAZ), leading to transactivation of MAZ and transcriptional alterations of its host gene CUX1 and other genes associated with tumor progression, thus promoting aerobic glycolysis and malignant progression of neuroblastoma [36]. In addition to the above two circRNAs, FECR1, a circRNA identified in the FLI1 promoter chromatin complex, was found to induce DNA demethylation by recruiting TET1 demethylase to bind to the promoter region of its host gene FLI1. Moreover, FECR1 also bound and downregulated DNA methyltransferase DNMT1, activating FLI1 transcription by inducing DNA hypomethylation of the promoter CpG islands, thereby promoting the invasion ability of breast cancer cells [35].

In addition to recruiting proteins to promote the transcription of host genes, some circRNAs can also inhibit the transcription of their host genes by sponging and binding RNA-binding proteins, thereby inhibiting the occurrence and progression of tumors. For example, circ-HUR was found to be down-regulated in gastric cancer tissues and cell lines, and interacted with the RGG domain of CCHC-type zinc finger nucleic acid binding protein (CNBP) to restrain its binding to the HuR promoter, thereby inhibiting the transcription of HuR, resulting in the down-regulation of its host gene HuR and repression of gastric cancer growth and aggressiveness in vitro and in vivo [82]. In summary, circRNAs can transcriptionally activate or inhibit the expression of their host genes by recruiting proteins, which is a critical mechanism of circRNAs involved tumorigenesis and tumor progression.

#### Regulating the expression of their host genes by forming an R-loop

R-loops are specialized chromatin structures, consisting of an RNA-DNA hybrid and a displaced single-stranded DNA, which are usually generated by RNA polymerase pause or RNA biogenesis dysfunction [99, 100]. R-loops have been shown to play critical roles in genome stabilization, and in general, R-loops may interfere with DNA replication, repair and transcription [101]. Recent studies show that circRNAs can increase the cleavage efficiency of homologous exon-defective mRNA by forming DNA hybrids or R-loops, which not only affects linear transcript abundance but also provides an mRNA trap to suspend transcription and improve splicing factors, which is also a critical mechanism of circRNAs to regulate host genes [94, 95]. So far, only circSMARCA5 has been found to regulate host gene expression through R-loop formation during tumor development. For example, Xu et al. found that circSMARCA5 was recruited to its host gene SMARCA5 locus to form an R-loop, which in turn terminated transcription, produced a truncated nonfunctional ΔSMARCA5 protein, and reduced the expression of SMARCA5 in breast cancer [87]. As SMARCA5 is a member of the SWI/SNF chromatin remodeling complex, which can be recruited to DNA damage sites during the process of DNA damage repair to induce the ubiquitination and phosphorylation of histone H2A, and promote chromatin remodeling and DNA damage repair [102]. Therefore, circSMARCA5 inhibits the expression of its host gene by forming an R-loop, which leads to a decrease of the DNA damage repair ability of its host gene and an improvement of the sensitivity of breast cancer cells to cytotoxic drugs, thus providing evidence that circSMARCA5 may be a therapeutic target for drug-resistant breast cancer patients (Fig. 1C). We believe that the regulation of host genes expression by circRNAs through R-loops formation will play a critical role in deciphers the mechanisms of tumorigenesis and progression in the future.

### Regulation at the post-transcriptional level

#### MicroRNA sponges

Competitive endogenous RNAs (ceRNAs) are transcripts that can regulate target genes at the post-transcriptional level through competitively binding to the shared miRNAs [103], which is also an essential way for circRNAs to participate in post-transcriptional regulation of target genes [104–106]. Several studies have demonstrated that circRNAs can bind to miRNAs as ceRNAs [26, 104–108], since miRNAs have an inhibitory effect on their target genes, the sponges and binding of miRNAs by circRNAs will lead to the upregulation of miRNA target genes, increase the expression of protein-coding genes, and then participate in the regulation of specific cellular pathways. Therefore, circRNAs may promote or inhibit tumor progression by indirectly regulating mRNA translation [109–111].

**Fig. 2:**
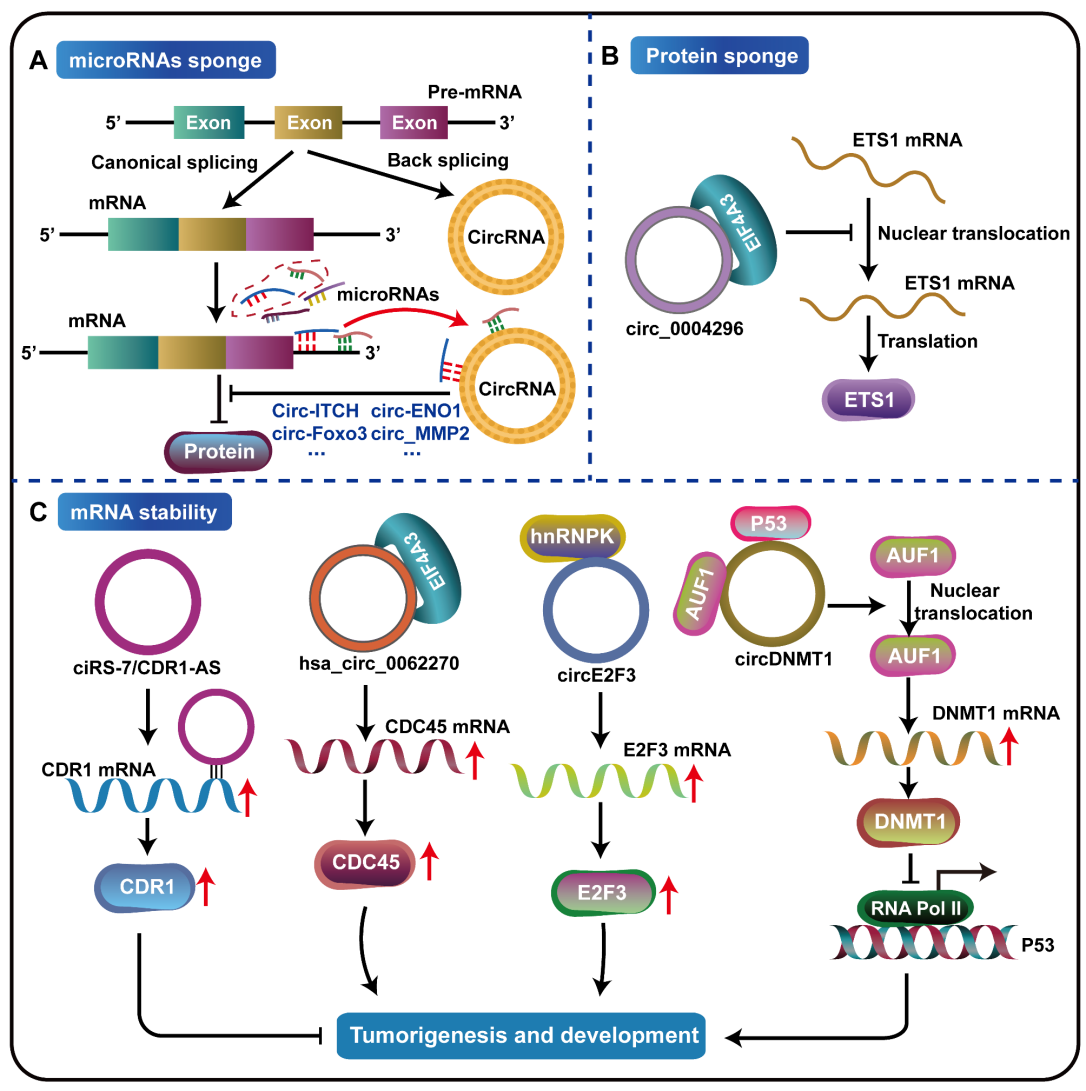
CircRNAs regulate the post-transcriptional modification of their host genes. **A** CircRNAs act as competing endogenous RNAs (ceRNAs) to relieve the adsorption of miRNAs on host genes and indirectly regulate the expression of their host genes. **B** CircRNAs, such as circ_0004296, function as protein sponges or decoys to regulate host gene expression and participate in the change of tumor malignant phenotype through post-transcriptional regulation. **C** CircRNAs enhance the stability of the host gene mRNA or induce the instability of the mRNA by directly interacting with the host gene mRNA or binding to RNA-binding proteins

Previous studies have suggested that circRNAs and their host genes contain one or more of the same microRNAs binding sites [25, 44, 78, 79]. Therefore, circRNAs can remove the inhibitory effect of microRNAs on their host genes by binding the shared microRNAs, and then participating in tumor growth and metastasis (Fig. 2A). For example, Li and others have shown that circ-ITCH shared the same miRNA binding sites with the 3’-untranslated region (3’-UTR) of the transcript from its host gene ITCH, and that circ-ITCH increased the expression of its host gene ITCH by sponging several miRNAs including miR-7, miR-17, and miR-214, thus inhibiting the Wnt/β-catenin pathway and the proliferation of esophageal squamous cell carcinoma cells and tumor growth in vivo by promoting ubiquitin-mediated Dvl2 degradation and decreasing the expression of oncogene c-Myc [26]. Circ-ENO1, also acting as a ceRNA, interacted with miR-22-3p to upregulate the expression of its host gene ENO1, and promoted glycolysis and tumor progression in lung adenocarcinoma (LUAD) [22]. Liu et al. found and confirmed that circ_MMP2 functions as a ceRNA to sequester miR-136-5p, and then positively regulated the expression of its host gene MMP2, which is transmitted to living cells in adjacent tissues through secreted exosomes, ultimately promoting the metastasis of hepatocellular carcinoma (HCC) [32]. In addition to the circRNAs mentioned above, many circRNAs as shown in Tables 1 and 2 can also establish circRNA-miRNA-host gene networks to participate in tumorigenesis and tumor progression in different tumors. In addition, a large number of circRNAs have been reported to regulate the expression of non-parental target genes by acting as ceRNAs and participate in the occurrence and development of tumors [104, 105], such as circEZH2/miR-133b/IGF2BP2/CREB1 [112], circBCAR3/miR-27a-3p/TNPO1 [113], which is also a critical mechanism of circRNAs involved in the cancer development.

#### Protein sponges

RNA-binding proteins also play a key role in post-transcriptional regulatory processes associated with different biological activities [114], and increasing evidence shows that circRNAs can act as protein sponges or decoys to participate in tumorigenesis and tumor progression through post-transcriptional regulation by binding to RNA-binding proteins to form a complex [88, 115] (Fig. 2B). Mao et al. found that the expression of circ_0004296, which is derived from back-splicing of exons 4, 5, 6, and 7 of host gene ETS1, was decreased in prostate cancer tissue, blood and urine. In addition, circ_0004296 was identified to be mainly distributed in the nucleus and interacted with the RNA-binding protein EIF4A3 to promote the retention of EIF4A3 in the nucleus and effectively inhibit the nuclear export of its host gene ETS1 mRNA, leading to the downregulation of ETS1 expression, thereby significantly suppressing proliferation, migration, invasion and EMT of prostate cancer (PCa) cells [88]. Altogether, circRNAs act as protein sponges to regulate the binding between proteins and nucleic acids and thus achieve certain biological functions.

#### mRNA stability

CircRNAs also can enhance the stability of the host genes mRNAs or induce the instability of the mRNAs by binding to RNA-binding proteins or directly interacting with the host genes mRNAs (Fig. 2C). It has been reported that circular RNA ciRS-7/CDR1-AS enhances the expression of the host gene CDR1 by directly interacting with the host gene to stabilize the mRNA of CDR1 [116]. In addition to circRNAs that can directly bind to the mRNAs of the host genes, studies have found that a variety of circRNAs, such as hsa_circ_0062270 [55], circE2F3 [40] and circDNMT1 [33], can regulate the stability of host gene mRNA by interacting with RNA-binding proteins, thereby participating in the tumorigenesis and development of tumors. For example, the study demonstrated that hsa_circ_0062270 was significantly upregulated in melanoma cells and could interact with RNA-binding protein EIF4A3 to positively regulate the expression of CDC45 by enhancing the stability of its host gene CDC45 mRNA, thereby promoting the proliferation, invasion and inhibiting the apoptosis of melanoma cells [55]. The study by Zhao et al. reported that circ_0075804 was upregulated in retinoblastoma (RB), which improved the stability of its host gene E2F3 mRNA and promoted the proliferation of RB by binding to the nucleic acid binding protein heterogeneous nuclear ribonucleoprotein K (HNRNPK) [40]. Circ-DNMT1 was reported to interact with both p53 and AUF1 (AU-rich element-binding factor 1) and promote the nuclear translocation of both proteins, and nuclear translocation of p53 induced autophagy, while nuclear translocation of AUF1 increased the stability of DNMT1 mRNA, leading to an increased translation of DNMT1, which ultimately increases the proliferation of breast cancer cells by stimulating cellular autophagy [33]. Taken together, circRNAs can regulate the expression of their host genes by promoting or inhibiting mRNA stability.

### Regulating the translation process of their host genes

**Fig. 3:**
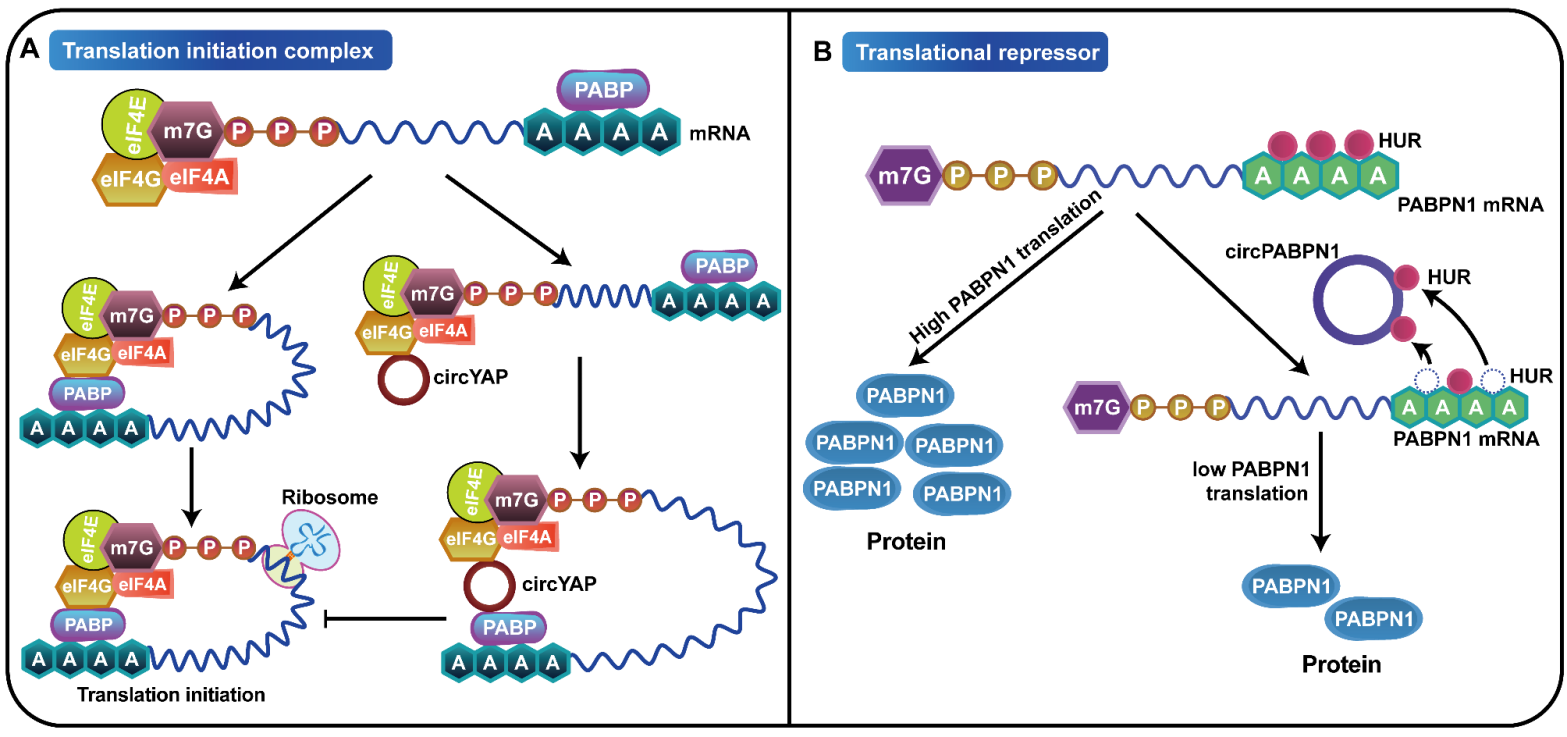
CircRNAs regulate the translation process of their host genes. **A** Some circRNAs, such as circYap, regulate the translation process of host genes by binding to translation initiation related proteins, increasing or decreasing protein synthesis. **B** Some circRNAs, such as circPABPN1, act as translation inhibitors or activators to regulate the binding of RNA-binding proteins to the mRNA of host genes, inhibit or promote the translation process of host genes, and affect the synthesis of proteins

The translation of messenger RNA into protein and the folding of the resulting protein into an active form is one of the most complex processes in the cell. The complex nature of this process makes it susceptible to deregulation at multiple levels. Studies have shown that circRNAs can regulate the translation process of host genes by binding to translation initiation-related proteins, thus increasing or decreasing protein synthesis, which in turn leads to tumorigenesis or progression (Fig. 3). YAP is a key component of the Hippo pathway [117, 118]. Inhibition of YAP activity could promote apoptosis, and inhibit proliferation and metastasis of tumor cells, suggesting that YAP as an important oncoprotein participates in the occurrence and development of tumors [119–121]. Wu et al. showed that circYAP was downregulated in breast cancer cells, which played a tumor suppressor role and significantly reduced YAP protein levels but had no effect on its mRNA levels. CircYAP was further found to bind with YAP mRNA and translation initiation related proteins eIF4G and PABP (poly(A) binding protein), which abolished the interaction of PABP on the poly(A) tail and eIF4G on the 5’-cap of the YAP mRNA translation initiation complex, and thus circYAP functions as a tumor suppressor gene by functionally inhibiting the translation initiation process of its host gene YAP [84] (Fig. 3A).

On the other hand, circRNAs can act as translation inhibitors or activators to regulate the binding of RNA-binding proteins to the mRNA of their host genes, thus inhibiting or promoting the translation process of their host genes. Abdelmohsen and colleagues [85] found that circPABPN1 was a circRNA derived from its host gene PABPN1, and PABPN1 was confirmed to be the target of HuR, which positively regulates PABPN1 protein translation by binding to PABPN1 mRNA. Furthermore, circPABPN1 inhibited the binding of HuR to PABPN1 mRNA, and therefore circPABPN1 reduced the translation of its host gene PABPN1 mRNA by competing with the translation activator (HuR), thus leading to metabolism disorders and tumorigenesis [85]. Therefore, we summarized the pathogenesis of circRNAs involvement in tumorigenesis by affecting translation dysregulation of host genes and described how translation dysregulation generates the phenotypic variability observed in tumors (Fig. 3B).

### Regulating the post-translational modification of their host genes

**Fig. 4:**
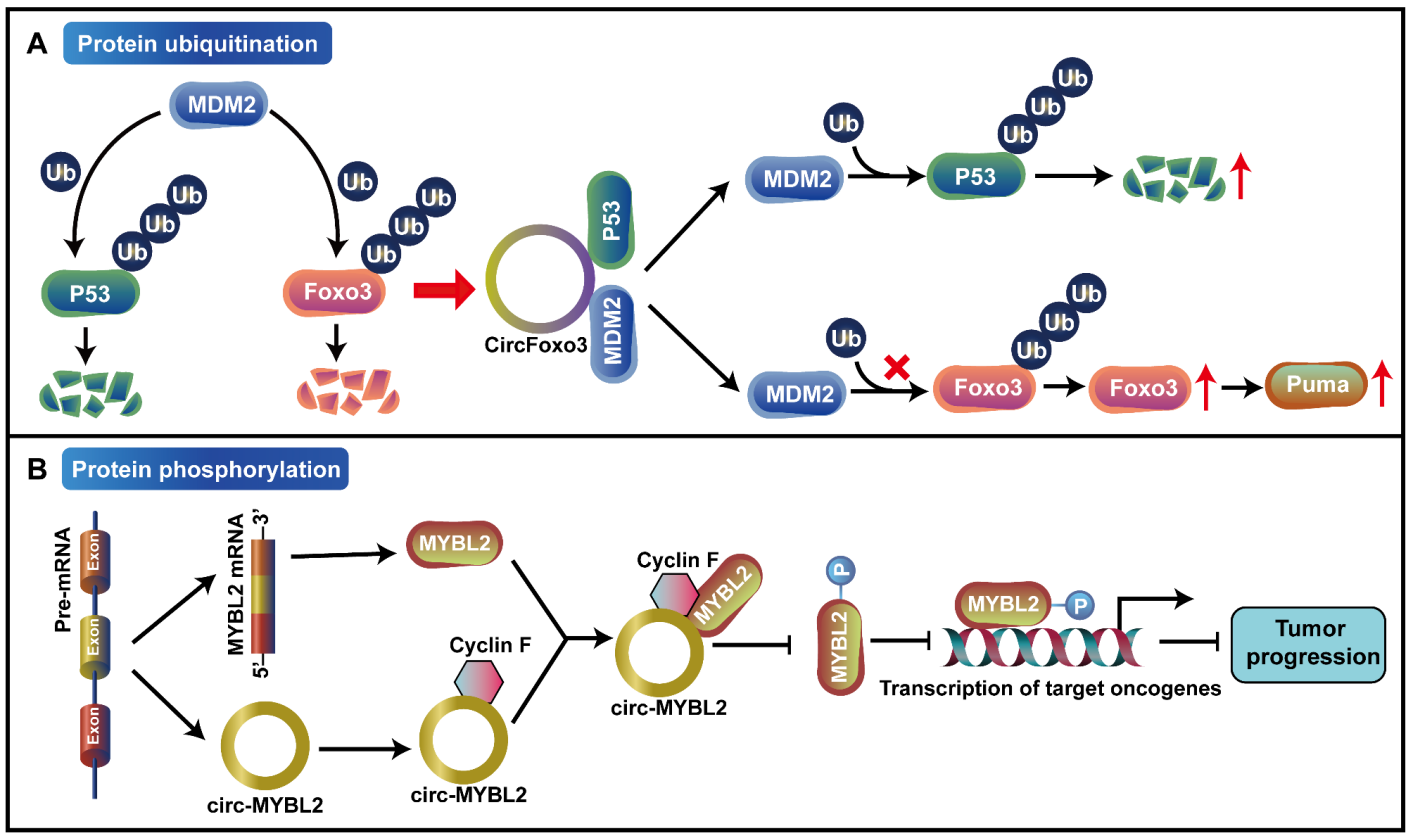
CircRNAs regulate post-translational modification of their host genes. **A** Some circRNAs, such as circFoxo3, regulate the activity and degradation of host proteins by recruiting proteins to regulate the ubiquitination of host proteins. **B** CircRNAs, such as circ-MYBL2, regulate the activity and degradation of host proteins by directly interacting with them to regulate the phosphorylation of host proteins

Post-translational modifications are essential for protein activity and degradation, such as acetylation, ubiquitination or deubiquitination and phosphorylation [122, 123]. Some studies have shown that circRNAs may regulate the activity and degradation of parental proteins by directly interacting with them or by recruiting proteins to regulate the post-translational modifications of parental proteins (Fig. 4). For example, Foxo3 gene is downregulated in many tumors and is considered as a tumor suppressor [124]. CircFoxo3 is a circular RNA spliced from Foxo3. Previous studies have shown that MDM2 can poly-ubiquitinate p53 and Foxo3 and down-regulate their expression in a proteasome-dependent manner [125]. Therefore, MDM2 plays a vital role in inhibiting apoptosis by inhibiting p53, Foxo3 and their downstream molecule Puma. Du et al. showed that circFoxo3 may interact with both p53 and MDM2 to promote MDM2-induced p53 ubiquitination and subsequent degradation in breast cancer, and avoid MDM2-induced Foxo3 ubiquitination and degradation [126]. Therefore, circFoxo3 promoted the expression of Foxo3 protein as well as its downstream target PUMA, thus inducing cell apoptosis [126] (Fig. 4A). At present, multiple myeloma (MM) is still an incurable disease, and revealing its pathogenesis will provide new targets for clinical diagnosis and treatment. Circ-MYBL2 was reported to be downregulated in multiple myeloma tissues, which could inhibit the phosphorylation and activation of its host gene encoding protein MYBL2 by promoting the binding of Cyclin F to MYBL2, thereby inhibiting the transcription of some critical proliferation-related oncogenes, and playing a tumor suppressor role [86] (Fig. 4B). Whether circRNAs can regulate other post-translational modifications in addition to the ubiquitination and phosphorylation of the parental proteins to regulate the expression of the host genes remains to be further explored.

### Regulating the expression of their host genes by encoding polypeptides

**Fig. 5:**
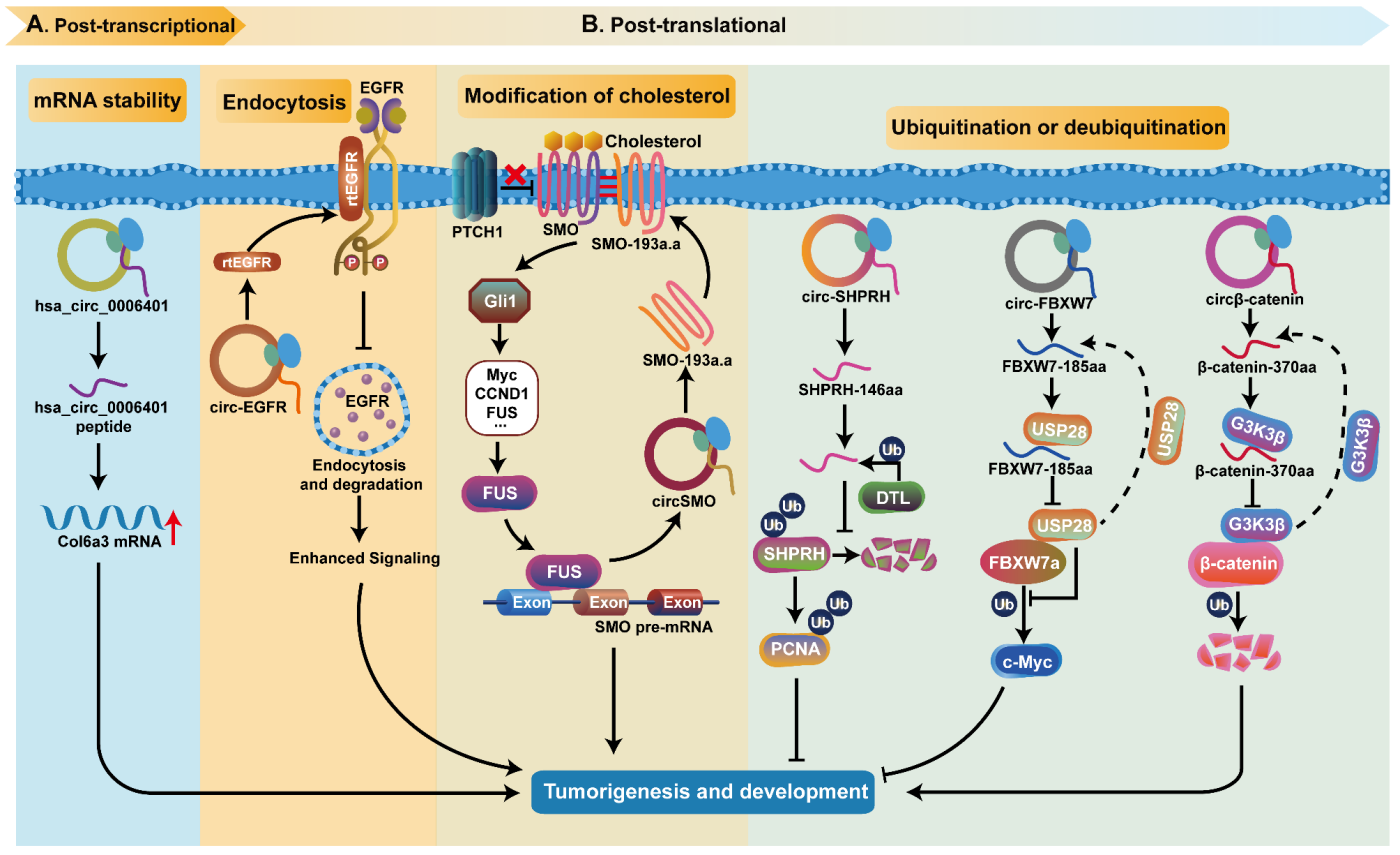
CircRNAs regulate their host genes expression by encoding polypeptides. **A** Small peptides encoded by circRNAs regulate the expression of host genes at the post-transcriptional level, thus participating in the malignant phenotype of tumors. **B** Small peptides encoded by circRNAs regulate endocytosis and degradation, cholesterol modification, ubiquitination and deubiquitination of host proteins at the post-translational level to regulate the stability of host proteins

For a long time, circRNAs have been thought to be directly involved in various biological processes as non-coding RNAs. In recent years, a variety of circRNAs have been found to have translation functions, and their encoded peptides have different functions similar to or opposite to the parental proteins, and also play biological roles in the occurrence and progression of tumors. Previous studies have demonstrated that the proteins encoded by circRNAs may regulate the stability of the host gene mRNA or host proteins at the post-transcriptional and post-translational levels (Fig. 5).

#### Regulation at the post-transcriptional level

Several studies have confirmed that small peptides encoded by circRNAs can regulate the expression of host genes at the post-transcriptional level, and then participate in the malignant phenotype of tumors. For example, Zhang et al. revealed that the hsa_circ_0006401 generated from col6a3 that contains an open reading frame (ORF) and encodes a novel 198-aa functional peptide, and the encoded hsa_circ_0006401 peptides could promote the stability of the host gene col6a3 mRNA at the post-transcriptional level, thereby promoting colorectal cancer (CRC) proliferation and metastasis [50] (Fig. 5A).

#### Regulation at the post-translational level

In addition to the regulation at the post-transcriptional level, more and more studies have shown that proteins encoded by circRNAs can also regulate endocytosis and degradation, cholesterol modification, ubiquitination and deubiquitination of host proteins at the post-translational level, thus to regulate the stability of host proteins, and then participate in tumorigenesis and progression (Fig. 5B). Liu et al. found that circ-EGFR can encode a polymetric novel protein complex, called rolling-translated EGFR (rtEGFR). When rtEGFR co-localized with EGFR on the cell membrane, rtEGFR directly interacted with EGFR to maintain EGFR stability and membrane localization, and attenuated EGFR endocytosis and degradation. Therefore, abnormal activation of the EGFR signaling pathway promoted the malignant progression of glioblastoma (GBM) [21].

In addition, some circRNAs can regulate cholesterol modification of proteins encoded by their host genes at the post-translational level. SMO-193a.a is a nascent protein with 193 amino acids generated from circSMO (hsa_circ_0001742), which is crucial for the Hedgehog (HH) signaling pathway [127]. Cholesterol modification is essential for full-length smoothened (SMO) activation, while PTCH1 in the HH signaling pathway can block SMO cholesterol modification [128, 129]. The authors further found that SMO-193a.a directly binds to the N-terminal of SMO, acts as a scaffold to transport cholesterol to full-length SMO, promotes cholesterol modification of full-length SMO, and releases SMO by inhibiting PTCH1, functionally maintaining the self-renewal ability of cancer stem cells and the tumorigenicity of GBM [127].

Another typical function of circRNAs is that they can regulate the ubiquitination and deubiquitination of their host genes encoding proteins at the post-translational level [29, 37, 68]. For example, Zhang et al. found that circ-SHPRH translated a new protein of 146-aa by overlapping genetic codes in glioblastoma. Both SHPRH and SHPRH-146aa can be used as ubiquitin targets of DTL, and SHPRH-146aa has a strong affinity. Mechanistically, SHPRH-146aa acts as a decoy to competitively bind DTL to protect the host SHPRH from degradation by the ubiquitin-proteasome [68]. Stabilized SHPRH, as an E3 ligase, ubiquitinates proliferating cell nuclear antigen (PCNA) [130, 131], thereby inhibiting cell proliferation and tumorigenicity [68]. Yang et al. reported that FBXW7-185aa is a new protein with 185 amino acids encoded by circ-FBXW7 [29]. So far, three FBXW7 isoforms, FBXW7a, b and c, have been reported, and the N-terminus of these isoforms is capable of being driven by the isoform-specific promoter [132]. The deubiquitination enzyme USP28 reportedly binds to the N-terminus of FBXW7a for deubiquitinating degradation, and then induces c-Myc to promote the development of GBM [29, 133]. Although FBXW-185aa is shorter than the above three subtypes, it has a stronger affinity to USP28 and binds to USP28 as a decoy, thereby inhibiting the proliferation of glioblastoma by releasing FBXW7a and reducing the half-life of c-Myc [132]. In addition to the two circRNAs mentioned above, the protein encoded by circβ-catenin, β-catenin-370aa, also promoted the growth of HCC cells by ubiquitination modification of its parental protein [37].

In summary, the discovery of these circRNAs and their encoded peptides enriches genomics and helps us to study the causes of tumorigenesis. The complex regulatory networks between the circRNAs encoded peptides and their host genes provide a new direction for the discovery of biomarkers for tumor diagnosis, prognosis and therapeutic targets.

## Regulatory network of circRNAs and their host genes

### Diversity of host genes regulation by circRNAs

In recent years, circRNAs have been reported to play dual functions in different types of tumors through different mechanisms, among which the regulation of circRNAs on their host genes is an important mechanism for participating in tumorigenesis and tumor progression [16, 25, 68, 69, 134]. For example, circ-Foxo3 functions as a tumor suppressor gene by positively regulating the expression of its host gene Foxo3 in breast cancer and non-small cell lung cancer [16, 25], however, it functions as an oncogene through a circ-Foxo3-miR-143-3p-USP44 axis independent of its host gene in gastric carcinoma [134]. Moreover, in recent years, the same circRNA could simultaneously regulate the expression of host genes through multiple mechanisms in the same tumor, supporting the specific and complex regulation of circRNAs on their host genes. For example, circ-CCND1 could not only combine with HuR protein to enhance the stability of CCND1 mRNA, but also act as a sponge for miR-646 to alleviate the inhibitory effect of miR-646 on CCND1 mRNA. Therefore, circ-CCND1 promotes the tumorigenesis of laryngeal squamous cell carcinoma (LSCC) by increasing mRNA stability and expression of CCND1 at the post-transcriptional [41]. CircMMP9 could interact with both AUF1 and miR-149, and block the inhibitory effect of AUF1 and miR-149 on the 3’-UTR of MMP9 to enhance the stability of MMP9 mRNA, thereby promoting the metastasis of oral squamous cell carcinoma [42]. FBXW7-185aa encoded by circFBXW7 inhibits the proliferation and migration of triple-negative breast cancer (TNBC) cells by increasing the abundance of FBXW7, inducing c-Myc degradation [30], and acting by the same mechanism as in glioblastoma, which has been described above [133]. Moreover, circFBXW7 could also upregulate the expression of FBXW7 by sponge of miR-197-3p to inhibit the progression of TNBC [30]. The above results show that, circRNAs function as oncogenes or tumor suppressor genes largely depending on tissue or cell type due to the diversity of target genes and mechanisms regulated by circRNAs.

**Fig. 6:**
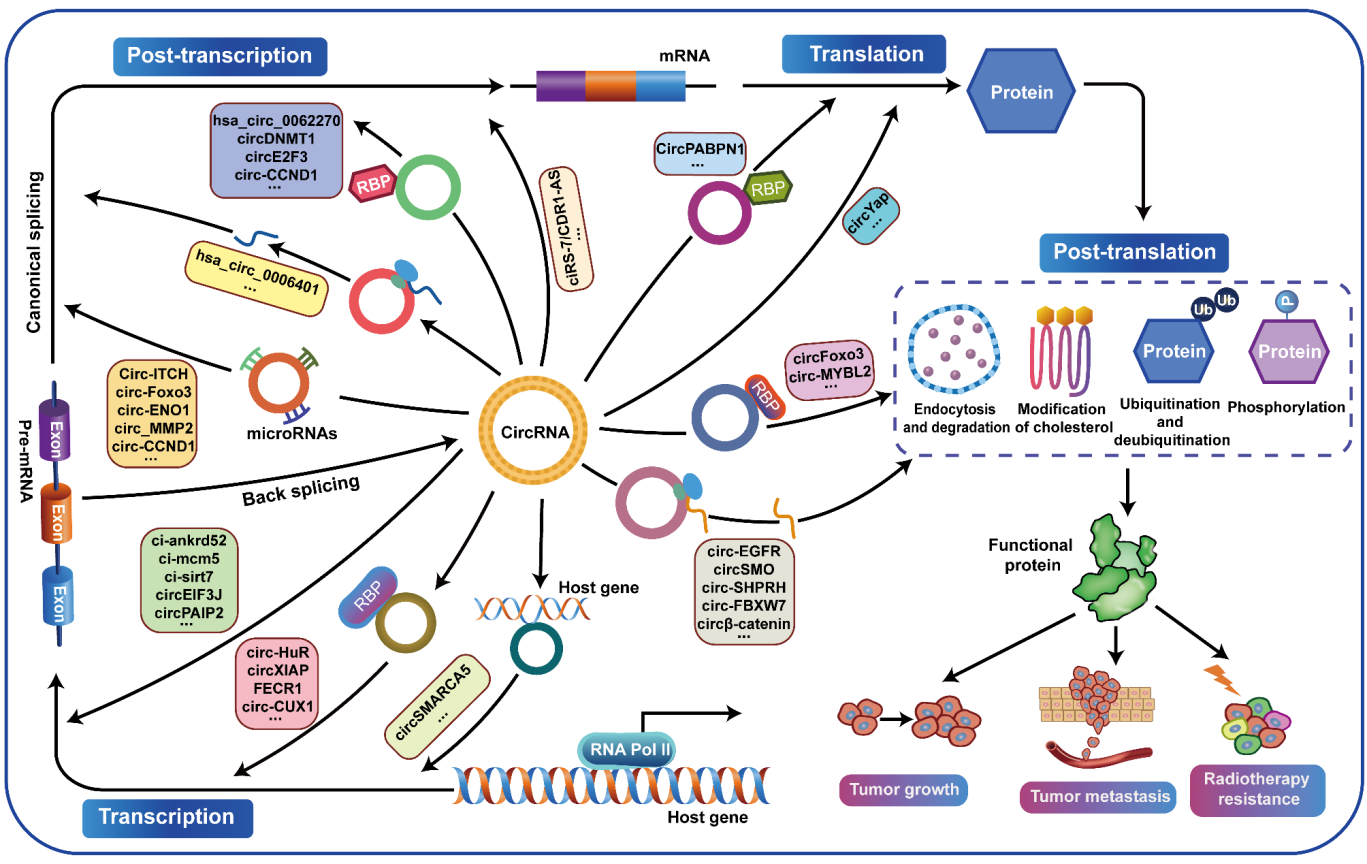
Regulatory network of circRNAs and their host genes. Schematic diagram of the molecular mechanism by which circRNAs regulate the expression of host genes and then participate in the tumorigenesis and development at transcriptional, post-transcriptional, translational, and post-translational levels

In conclusion, circRNAs regulate the expression of their host genes through a variety of mechanisms at the transcriptional, post-transcriptional, translational, and post-translational levels, which forming a complex network to reveal the mechanisms of tumor malignant progression (Fig. 6).

### A complex regulatory network between circRNAs and their host genes

As shown in Tables 1 and 2, we enumerated some circRNAs with the same and opposite functions as their host genes, and detailed the mechanisms by which circRNAs regulate the expression of the host genes at the transcriptional, post-transcriptional, translational, and post-translational levels. The production of circRNAs can affect the accumulation of linear mRNA, thus regulating genes expression [33, 40, 50, 55]. Therefore, the regulation between circRNAs and the host genes not only affects the linear transcript abundance of the host gene, but also provides a feedback loop that can regulate the formation of circRNA. For example, circMbl is derived from exon 2 of MBL and can directly bind to MBL, and MBL is prevented from binding to other targets. Moreover, MBL can also interact with flanking introns to regulate the formation of circMbl [135]. The regulation of MBL levels strongly affects the biosynthesis of circMbl, which is dependent on MBL binding sites, forming a positive feedback network.

CircRNAs are formed by back splicing of precursor mRNAs. Similarly, pre-mRNA requires further splicing modification to form mature mRNA after transcription [136]. For most host genes, the production of circRNAs is usually incompatible with functional mRNA formation, and there is a passive competition between them, with circRNAs production coming at the expense of a reduction in its corresponding mRNA isoform. Under certain circumstances, such as when pre-mRNA processing is slowed down, the nascent RNA can be directed to alternative pathways that promote back-splicing [137–139]. On the other hand, some circRNAs can compete with linear alternative splicing (AS) targets, and logically, back-splicing is less efficient than canonical splicing due to suboptimal spliceosome assembly at the back-splicing site. However, due to core damage, which refers to the depletion or pharmacological inhibition of core spliceosome components that control the RNA outputs of reporter and endogenous genes, splicing factors were inhibited, leading to the suppression of pre-mRNA splicing and enhancement of back-splicing [140–142]. Comparison of back-splicing and linear splicing further suggests that although splicing factors can control both processes, the splicing regulation rules of circular RNA biogenesis are different from those of linear splicing [143]. In addition, it has been proposed that linear splicing and back splicing may compete for limited splicing factors, introducing flanking exons with strong 5’ and 3’ splice sites, greatly reducing looping efficiency [135], and in addition to canonical splicing signals, important signal sequences in the spliceosome machinery (such as polypyrimidine tracts) also affect looping. Therefore, the abnormal increase and decrease of circRNAs will break the balance between the linear host genes and the circRNAs, forming a double negative or double positive feedback regulatory loop to regulate the expression of the host genes (Fig. 6).

## Combination of circRNAs and their host genes is a potential molecular target for tumor diagnosis and treatment

The lack of effective diagnostic markers and therapeutic targets in tumor patients is part of the reason for their poor prognosis. Therefore, it is urgent to find biomarkers or therapeutic targets to improve the clinical prognosis of tumors. CircRNAs have been proved to have great potential in tumor diagnosis and prognostic biomarkers, and are becoming an emerging field of tumor diagnosis and treatment research [144–146].

### Combination of circRNAs and their host genes might be a set of biomarkers for tumor diagnosis and prognosis

The expression patterns and characteristics of circRNAs make them ideal biomarkers. Firstly, circRNAs are highly stable and have a long half-life due to their circular structure, which makes them more resistant to RNase R exonuclease degradation than the corresponding linear RNAs. This stability makes circRNAs more easily detectable and thus are applied to clinical diagnosis [12, 20, 33]. Secondly, the expression of many circRNAs is tissue-specific and developmental stage specific, which plays an important role in diagnosis and prognosis. Moreover, circRNAs have been reported to perform their biological functions inside cells, or can be identified in human blood and urine through exosomes export, used for non-invasive detection [2, 147–149], and to be taken up by adjacent (paracrine) or distant cells (endocrine), and affect many aspects of the physiological and pathological conditions of recipient cells [150, 151].

The study showed that circITGA7 and ITGA7 were low expressed in colorectal cancer tissues. The receiver operating characteristic (ROC) curve analysis, which is the most popular graphical method for evaluating the classification accuracy of a diagnostic marker [152–154], showed that the area under the curve (AUC) of circITGA7 was 0.8791 with a sensitivity (true-positive rate = true positives/[true positives + false negatives]) of 0.9275 and a specificity (true-negative rate = true negatives/[true negatives + false positives]) of 0.6667, which was much higher than that of ITGA7 (AUC = 0.7402) [18]. AUC (takes values from 0 to 1) is an effective way to summarize the overall diagnostic accuracy of the test. Generally, the higher AUC test may be considered better [155, 156]. In conclusion, circITGA7 has the potential as a biomarker for the diagnosis of colorectal cancer. In addition, it was also found that the expression level of circITGA7 was negatively correlated with tumor size, lymph node metastasis, distant metastasis and TNM stage [18]. The study found that circZKSCAN1 and linear ZKSCAN1 were low expressed in liver cancer tissues, and the area under the curve (AUC) of cirZKSCAN1 was 0.834, with a sensitivity of 82.2% and specificity of 72.4%, which was much higher than that of ZKSCAN1. In addition, it was found that among all clinical parameters, the low expression level of ZKSCAN1 was correlated with tumor size [19]. In addition to circITGA7 and circZKSCAN1, there are many circRNAs, such as circGSK3β [80], circ-CCNB1 [83], circ_MMP2 [32], circ-ITCH [17], circCOL6A3 [31] and circ-SHPRH [68] are also abnormally expressed in tumor tissues, which are related to the occurrence and progression of tumors and can be used as biomarkers for clinical diagnosis and prognosis.

### Combination of circRNAs and their host genes is a set of potential molecular targets for cancer therapy

Although there are no clinical reports of circRNAs for targeted therapy, their low molecular weight, stability, conservation, and regulatory effect on tumor cell activity make it possible to become a molecular drug or target for tumor therapy [157, 158]. With the gradual maturity of artificial circRNAs construction and circRNAs interference technology, it is possible to regulate circRNAs, which will provide a new way for tumor treatment.

In tumor progression, circRNAs stimulate or stabilize the expression of host genes through positive or negative feedback mechanisms, and then play a role in promoting or inhibiting the occurrence and development of tumors. Numerous studies have shown that in tumors, interactions between circRNAs and their host genes are involved in regulating the downstream pathways of host genes, increasing the richness and complexity of potential mechanisms. Therefore, linking the expression of circRNAs with the expression changes of host genes plays a role of signal amplification and is more helpful for clinical treatment. CircRNAs such as circ-ENO1 [22], circGFRA1 [39], circCCDC66 [64], circ-Amotl1 [24] and circ-Foxo3 [16] have been found to participate in tumorigenesis and metastasis by regulating the expression of host genes. Therefore, we anticipate that targeting the circRNAs/host genes regulatory axis will provide information for innovative therapeutic targets, indicating the important role of the regulatory networks of circRNAs as well as their host genes as biomarkers in tumors.

## Conclusions

With the continuous progress of the RNA field, circRNAs have become a new research hotspot. In recent years, a large number of studies have deepened our understanding of circRNAs, and their interaction with tumors has gradually attracted people’s attention. CircRNAs are derived from host genes, and in human tumors, similar to the regulatory effect of circRNAs on other targets, they can regulate the transcription, post-transcription, translation, protein activity and degradation of host genes. Emerging studies have demonstrated that circRNAs, as biomarkers or regulators, participate in human diseases and may improve clinical treatment in the future in combination with the currently widely used diagnostic and therapeutic methods. Because it is likely that the complex functional networks composed of circRNAs, rather than a single circRNA, affect tumorigenesis, a reasonable research advance should be to screen circRNAs and then investigate the function of a group or a single of significantly differentiated circRNAs. The combination of circRNAs and their host genes plays a role in signal amplification, which is helpful for later diagnosis and treatment, thus further exploration of circRNAs will help us better understand their heterogeneity.

## Data Availability

Not applicable.

## Abbreviations

CircRNAs: Circular RNAs
RBPs: RNA binding proteins
ncRNAs: Non-coding RNAs
ESCC: Esophageal squamous cell carcinoma
EMT: Epithelial-mesenchymal transition
pre-mRNA: Precursor mRNA
ecircRNAs: Exonic circRNAs
ciRNAs: Intronic circRNAs
EIciRNAs: Exon-intron circRNAs
Pol II: Polymerase II
EWSR1: EWS RNA-binding protein 1
MAZ: MYC-associated zinc finger protein
CNBP: CCHC-type zinc finger nucleic acid binding protein
ceRNAs: Competitive endogenous RNAs
3’-UTR: 3’-untranslated region
LUAD: Lung adenocarcinoma
HCC: Hepatocellular carcinoma
PCa: Prostate cancer
RB: Retinoblastoma
HNRNPK: Heterogeneous nuclear ribonucleoprotein K
AUF1: AU-rich element-binding factor 1
PABP: Poly(A) binding protein
MM: Multiple myeloma
ORF: Open reading frame
rtEGFR: Rolling-translated EGFR
SMO: Smoothened
GBM: Glioblastoma
HH: Hedgehog
PCNA: Proliferating cell nuclear antigen
AS: Alternative splicing
ROC: Receiver operating characteristic
AUC: Area under the curve
